# A neural encoder for earthquake rate forecasting

**DOI:** 10.1038/s41598-023-38033-9

**Published:** 2023-07-31

**Authors:** Oleg Zlydenko, Gal Elidan, Avinatan Hassidim, Doron Kukliansky, Yossi Matias, Brendan Meade, Alexandra Molchanov, Sella Nevo, Yohai Bar-Sinai

**Affiliations:** 1 Google Research, Tel-Aviv, Israel; 2 Google Research, Cambridge, MA USA; 3 Department of Earth and Planetary Sciences, Harvard University, Cambridge, MA USA; 4 Department of Condensed Matter Physics, Tel-Aviv University, Tel-Aviv, Israel

**Keywords:** Seismology, Seismology

## Abstract

Forecasting the timing of earthquakes is a long-standing challenge. Moreover, it is still debated how to formulate this problem in a useful manner, or to compare the predictive power of different models. Here, we develop a versatile neural encoder of earthquake catalogs, and apply it to the fundamental problem of earthquake rate prediction, in the spatio-temporal point process framework. The epidemic type aftershock sequence model (ETAS) effectively learns a small number of parameters to constrain the assumed functional forms for the space and time correlations of earthquake sequences (e.g., Omori-Utsu law). Here we introduce learned spatial and temporal embeddings for point process earthquake forecasting models that capture complex correlation structures. We demonstrate the generality of this neural representation as compared with ETAS model using train-test data splits and how it enables the incorporation additional geophysical information. In rate prediction tasks, the generalized model shows $>4\%$ improvement in information gain per earthquake and the simultaneous learning of anisotropic spatial structures analogous to fault traces. The trained network can be also used to perform short-term prediction tasks, showing similar improvement while providing a 1000-fold reduction in run-time.

## Introduction

The application of machine-learning (ML) for the analysis of seismological data has seen substantial recent progress highlighted by new approaches for the classification and characterization of seismic waveforms^1,2^, automatic phase picking^3^, identification of low-magnitude earthquakes^4^, and catalog declustering^5,6^. In the development of earthquake catalogs ML approaches have increased the number of detected events by ten folds^4^ and will possibly reduce travel time dependence for earthquake early warning from the speed of seismic waves to the speed of light^7^.

However, in earthquake sequence modeling machine learning techniques have yielded limited progress in terms of enabling improved characterizations seismicity patterns^8,9^. The specific task of forecasting the timing of future seismic events is a longstanding and fundamental challenge both as a basic scientific question and for applied hazard analysis. While in some cases seismic activity features relatively consistent temporal^10^ or spatial patterns^11^, the time, location and magnitude of seismicity has remained difficult to predict quantitatively^12^.

The state-of-the-art approach to this problem in statistical seismology is to represent earthquake sequences as a spatio-temporal point process^13–15^. In this approach, the model is tasked with predicting the instantaneous rate of earthquake occurrence above a certain magnitude, $\lambda (x, y, t \mid H_{t-})$, where *x*, *y* are spatial coordinates (longitude and latitude or map projected coordinates) and *t* is time. $H_{t-}$ represents all the information available to the model prior to time *t*. The time-dependent function $\lambda$ is the quantitative representation of the intensity of seismic activity, characterizing both the foreshock^16,17^ and aftershock^18^ epochs as well as serving as the foundation for seismic hazard assessment^19^.

The epidemic-type aftershock sequence (ETAS) model^13,20^ is the most commonly used such model, representing $\lambda$ as a self-exciting branching process, which assumes a “background rate” of seismicity and a response function, *f*, whose specific form is chosen such that the long-term statistics of synthetic earthquake catalogs generated from the model reproduce the two widely observed phenomenological distributions of seismicity: (1) the Omori-Utsu law of aftershock rate decay and (2) the Gutenberg-Richter distribution of event magnitudes. There are a few popular choices for the response function^21–24^, that share the form of, $f = \mu (x,y)+ T(t-t_i)S(x-x_i, y-y_i; M_i)$. Here $\mu$ is called the time-independent “background rate”, *T* is a temporal kernel featuring a power-law decay consistent with Omori’s law, and *S* is a spatially decaying kernel^22,25^. $x_i, y_i$, and $t_i$ are the earthquake’s hypocentral location and occurrence time, respectively.

The ETAS model has been used as an effective representation of earthquake rate changes^19,26–28^. However, its applicability has been limited by several factors. First, finding optimal ETAS parameters is a challenging optimization task, because of a broad minima associated with the the space-dependent background seismicity rate and a range of different parameters for the response function can produce similar log-likelihood scores^29–33^. Second, the classical predetermined forms of *f* have a limited expressive power and limit the ETAS approach to the consideration of the hypocenters, times, and magnitudes of past moderate-large magnitude earthquakes. Additional relevant data including small magnitude seismicity, tectonic structure, fault locations and earthquake focal mechanisms are typically not modeled, though some attempts have been made to incorporate them^19,21,34,35^.

**Figure 1:**
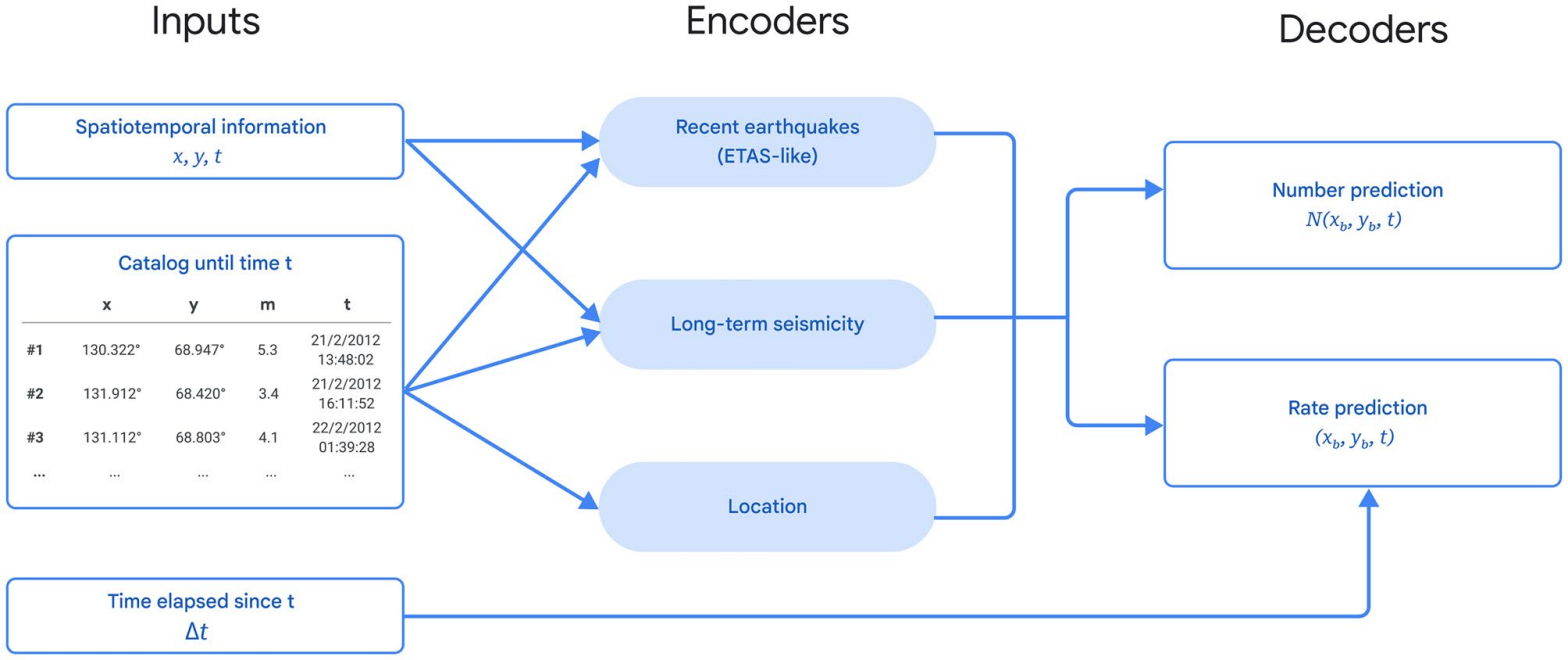
A sketch of the model architecture incorporating information up to time *t*.

Here we propose the FERN (Forecasting Earthquake Rates with Neural networks) encoder-decoder neural based model to generalize beyond the ETAS constraints. Conceptually, the input is encoded by a neural network to generate a latent representation of the tectonic state, which is then passed to a decoder network (Fig. 1). This design has two specific advantages: first, it naturally allows to incorporate different data sources and modalities, which can be added to the model with source-specific encoders. Second, the same encoded state can be used as input to several prediction heads (“decoders”), which can be used to for different prediction tasks.

This approach matches the performance of the state-of-the-art ETAS model in rate prediction when trained on identical data sets and that the FERN model exhibits increased accuracy when supplied with earthquakes of magnitude smaller than the completeness magnitude threshold of the catalog. We also show how the trained encoders can be used to solve a different prediction problem, a short-term forecast of the number of events in a 24-h period. In this task, the FERN model outperform the ETAS model while requiring 4-5 orders of magnitude less compute time. We do not provide any uncertainty estimates based either on either data error propagation or varying model architecture.

We use three encoders (Fig. 1) to capture different aspects of the seismicity patterns. The *recent earthquakes encoder* is a direct generalization of the ETAS response function *f*, replacing the human-engineered functional form of *f* by a more general neural network. It is intended to capture short-term seismic activity. The *long term seismicity encoder* learns long-term spatio-temporal seismic patterns by counting earthquake events in varying temporal spans, ranging from minutes to years. Lastly, the *location encoder*, analogous to the background rate of seismicity in the ETAS model, learns location-specific information. Details of the encoder architectures are given in the methods section below, and the source code is available at^36^.

## Results

Here we apply the FERN model to the observed seismicity of the greater Japanese Islands region recorded over the last 30 years. The study region is discretized into a grid of square cells of dimension $0.25^{\circ } \times 0.25^{\circ }$. The input to the model is a catalog of earthquakes, including the hypocenter, magnitude, and time of each event, as well as the geographic location of the gridded cell centers. This information is passed through three neural encoders to generate a latent representation of seismic history. The encoded history is then passed through a neural decoder to perform the prediction task.

**Figure 2:**
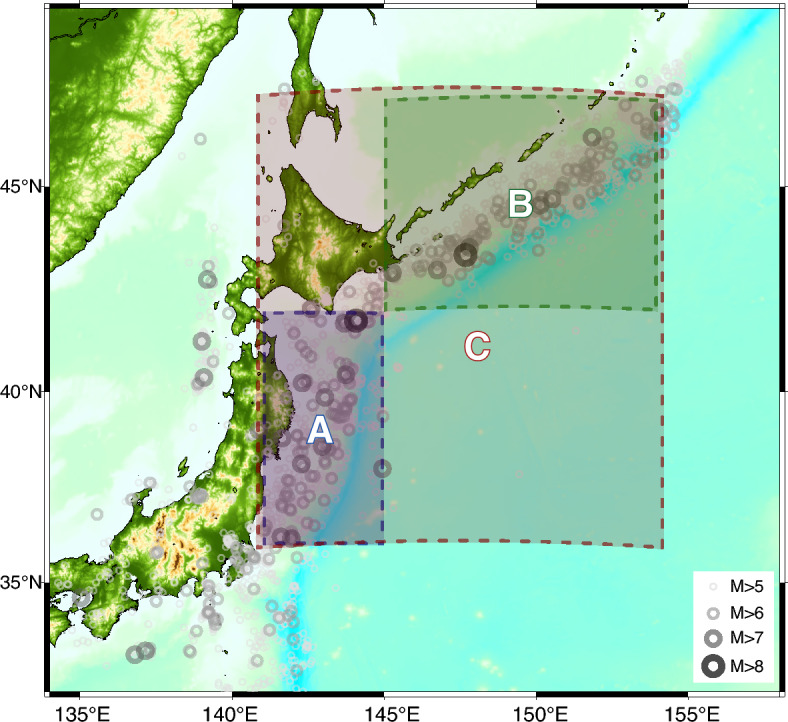
The three study regions in northern Japan. Earthquakes larger than $M_w=5$ that occurred during the study period are plotted. Maps, here and in Fig. 4, were generated using the pygmt^37^ and matplotlib^38^ python packages.

We apply the FERN model to study the seismic activity in three sub-regions near the Japan subduction zone (Fig. 2). Using hypocenter data from the JMA earthquake catalog^39^ the network is trained separately in each region using strict train-validation-test temporal splits of the data with a training period spanning the years 1979-1995 and a validation period of 1996–2003. A hyper-parameter search is performed to determine the optimal network parameters. Finally, the best performing model is trained over both the training and validation period, and is evaluated over the catalog of the years 2004–2011 (test period). The evaluation is performed over a finer grid, $0.05^{\circ } \times 0.05^{\circ }$, to obtain a better estimation of model performance. Numerical tests have demonstrated that further resolution refinement does not improve our estimation of the log-likelihood. All metrics reported below pertain to the performance of the FERN model during a test period that ends prior to the great Tohoku-oki earthquake of March 2011. Simultaneously, we also train an ETAS model^26,40–42^ over the same temporal and spatial windows. Average seismicity rates in the three period are given for each region in Table I of the supplementary material.

### Earthquake rate prediction

As a first step, we train the FERN model to predict the instantaneous rate of seismicity, $\lambda (x,y,t \mid H_{t-})$ which is also the output of the ETAS model. The network is trained to optimize the log-likelihood of the observed catalog, ${\mathscr {L}} = \sum _i \log \lambda _i-\iiint \lambda (x,y,t)dx\,dy\,dt$^13,15^ where $\lambda _i = \lambda (x_i, y_i, t_i \mid H_{t_i-})$ is the predicted rate at the spatiotemporal location of the *i*-th earthquake and the sum is taken over all earthquakes in the study region above a certain magnitude cutoff $M_c$ which we assume to be the estimated completeness magnitude of the catalog.

**Table 1:** Average Information Gain Per Earthquake (IGPE) for all models in the rate-prediction task.

		Train	Test
Region A	ETAS	3.280	2.278
	FERN	3.354	2.308
	FERN+	3.529	2.395
Region B	ETAS	2.892	2.616
	FERN	2.981	2.573
	FERN+	3.140	2.749
Region C	ETAS	1.877	1.395
	FERN	1.807	1.717
	FERN+	2.035	1.803

We find that in all three study regions FERN exhibits a comparable log-likelihood score to that of ETAS (Table 1). Because FERN enables the incorporation of additional information without modification of the model architecture, we can directly include potentially precursory seismic activity from earthquakes of magnitude lower than $M_c$ using these smaller events only as features, but not as labels. That is, the low-magnitude earthquakes are included as input to the model, but do not change the calculation of ${\mathscr {L}}$. This allows a proper statistical comparison of the model including smaller events (FERN+) with ETAS and with FERN, as all these models describe the same statistical space, namely seismicity above $M_c$. The additions of smaller magnitude seismicity improves the information gain per earthquake by 4-12% in all tested regions as compared to both ETAS and FERN with large earthquakes only (Table 1). This amounts to $\sim 0.1$ information bits per earthquake on average.

### Short-term forecasts

As a second test, we train the FERN model to perform a short-term seismic forecast. Using the same encoders that were trained to perform rate prediction, and without updating their weights, we now train a different decoder that performs a short term forecast for the number of earthquakes of magnitude $>M_c$ that occur in each spatial $0.5^{\circ } \times 0.5^{\circ }$ cell. Specifically, the features in each training example are the earthquakes that occurred up to time *t* and the label for each cell is the number of earthquakes that occurred in it in the 24 h after time *t*. Unlike rate prediction, this is a standard (supervised) regression problem whose metrics are readily interpretable. We follow the same strict train-validation-test split as above for training the decoders (the encoders are not retrained), and benchmark model results against catalogs generated from the trained ETAS model. We follow the standard protocol^26^ of generating 100,000 catalogs from ETAS for each day, and calculating the average number of earthquakes in each cell. The results are presented in Table 2.

We compare model performance using Receiver Operating Characteristic analysis (ROC) obtained by thresholding the model output and counting the rate of true positive (TPR) rate and false positive rate (FPR) predictions (TPR here means that at least one earthquake occurred within a grid cell during a target time interval). For example, in region C at a FPR of 20% ETAS provides a TPR of 80% while FERN+ shows a TPR of 90%. Similar results are obtained for region B, while in region A all models show similar performance.

**Figure 3:**
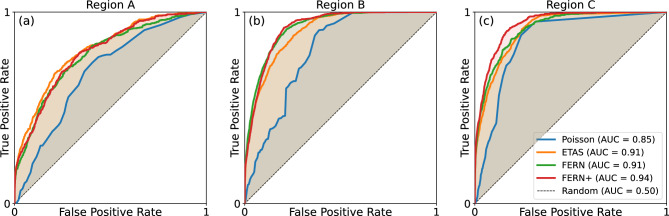
ROC curves for different models in region C. Regions A,B show qualitatively similar results.

This is also true in other statistical tests, as shown in panel (b) of Fig. 3. In it we compare the likelihood score of the observed seismicity in the test period (“L-test”) assuming the number of earthquakes in each cell follows a Poisson distribution, and the likelihood score when comparing only the spatial distribution of earthquakes over the test period (“S-test”), see supplementary material for more information. We note that performing short term prediction with FERN (or FERN+) requires only a single forward pass of the trained network, while an ETAS prediction requires running a large number of simulations to collect catalog statistics^26^. This means that FERN+ provides more than a 1000-fold improvement in runtime.

It should be noted that the performance of all models, both machine-learned and ETAS, varies across different geographical regions and time windows^43^, as we see here as well. For example, it is seen that the information gain of FERN+ over ETAS in Region A is relatively small. It is difficult, in general, to interpret why the neural model performs well in one region and less so in others, though we believe that in this case the cause is the change in seismicity statistics between the train+validation periods, on which the models were trained and calibrated, and the test period, for which the metrics are reported. Table I of the supplementary material details these statistics. It shows that region A shows much more $M_w\ge 7$ earthquakes in the test period (0.88 events/year) than in the train+validation period (0.2/year). Such a dramatic change does not occur in region B or C. Such effects might be mitigated by continuous training of the model (“pseudo-prospective testing”) or by training a model on several regions in parallel. However, it is worth noting that even in region A the neural model achieves comparable metrics to that of ETAS.

### Inspecting the trained model

**Figure 4:**
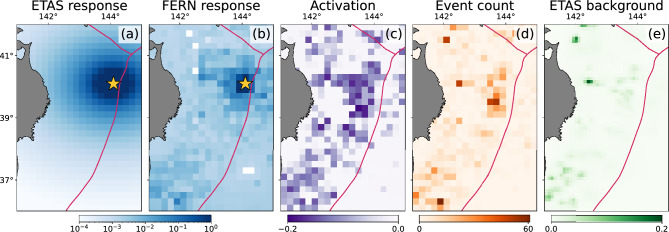
Looking inside the model. (**a**,**b**) The rate difference, as predicted by ETAS and FERN to a single earthquake. We added a synthetic earthquake to the catalog at $144^{\circ }, 40^{\circ }$ (marked with a yellow star) at time $t=10.10.2010$ at midnight, and calculated the difference between the rate predicted by the models with and without the synthetic earthquake, 1 h after the event. The plotted region is Region A, and the fault line is shown in red. (**c**) The activation of one of the latent neurons in the output of the location encoder, for each spatial cell (other neurons show qualitatively similar patterns). It is seen that this patterns correlates well with total number of earthquakes in the cell, shown in (**d**). We can think about the output of the location encoder as a generalization of the background rate $\mu$ of the ETAS model, which is shown in (**e**).

Unlike ETAS, the parameters of the neural model cannot be trivially interpreted, which is common for neural models^44^. However we can experiment with FERN model to answer the question: How does the predicted seismicity rate $\lambda (x,y,t)$ change in response to a single earthquake? The answer that the ETAS model gives to this basic question is, by definition, *f*. To answer this question with the FERN model, we added an synthetic earthquake to the event catalog, at an arbitrary time and location in Region A (cf. Fig. 2). In Fig. 4 we present the difference between model prediction for $\lambda (x,y,t)$ 1 h after this synthetic earthquake and its prediction when this earthquake is not present, for both ETAS and FERN.

We find that the response of FERN shows a complex and anisotropic spatial structure, with increased response along the fault trace. We note that the location of the fault line was not included as a feature to the model and that the FERN model learns that the increased seismic activity is neither isotropic nor spatially homogeneous which is, of course, a well known characteristic of seismicity^45–49^. It is also seen that the output of the location encoder shows similar spatial patterns to the patterns of seismic activity, as was recently shown^50^. Similarly we find that the temporal dependence of the the rate increase learned by FERN is a power-law, but one that decays slower than the ETAS prediction, depends less strongly on the magnitude, and the magnitude dependence is not homogeneous but rather spatially dependent (Supplementary material).

**Table 2:** Classification metrics for all models across all region in the short-term prediction task, evaluated on the test set.

Region	Log-likelihood score			S-test			AUC ROC			Runtime
	A	B	C	A	B	C	A	B	C	
Poisson	3553	6383	4847	513	1539	1243	0.69	0.79	0.85	$\sim 1 \, \upmu$ s
ETAS	2927	4993	3849	160	364	290	**0.80**	0.90	0.91	$\sim 10$ h
FERN	2967	4770	3701	166	**194**	235	0.78	**0.92**	0.91	$\sim 1$ s
FERN+	**2926**	**4747**	**3515**	**144**	209	**157**	0.79	**0.92**	**0.94**	$\sim 1$ s

Best metric for each experiment is typed in boldface. The log-likelihood score and AUC ROC (area under the ROC of Fig. 3) are standard classification metrics^44^. The S-test is a log-likelihood score disregarding the temporal distribution of earthquake times, commonly used in earthquake forecast evaluation^51,52^.

## Conclusions

We present a neural architecture for earthquake rate forecasting, adopting the point-process approach but replacing the assumed functional forms of the ETAS model with learned embeddings. Our method shows comparable or superior test metrics (without uncertainty analysis), and the latent representation of seismic history generated by the neural encoders, which were trained to perform rate prediction, can readily be used also for related tasks with small additional effort. This raises hope that such models could be useful in other tasks, such as magnitude prediction or hazard assessment.

## Methods

### Neural architecture

Here we describe the main design choices of the FERN model. Full details can be found in the supplementary material.

#### Encoders

1. *Recent earthquakes (ETAS-like):* This encoder model is a direct generalization the sum term in the definition of ETAS. That is, its output is a sum of a function applied to cataloged data of every earthquake in the (recent) past. The function is constructed in the following way: The catalog provides 5 numbers that describe each earthquake, indexed by *i*: the time of the event $t_i$, its epicentral location $x_i, y_i$, depth $d_i$ and moment magnitude $M_i$. We use UTM coordinates for *x*, *y*. In addition, the model has access to the spatiotemporal parameters of the cell *x*, *y*, *t*. For each earthquake and cell we calculate a list of *k* features $F^1(t,x,y,t_i,x_i,y_i,d_i,M_i)\dots F^k(t,x,y,t_i,x_i,y_i,d_i,M_i)$. These feature functions are inspired by ETAS and constrained by physical considerations. A few examples of feature functions are the magnitude of the earthquake, $F_1 = e^{M_i}$; the reciprocal of the elapsed time since the earthquake, $F^2 = 1 / (t - t_j)$; the reciprocal of the distance earthquake’s epicenter, $F^3= 1 / \sqrt{(x - x_j)^2 + (y - y_j)^2}$, etc. The full list of feature functions is given in table III of the supplementary material. The feature vector $\left( F^1_i,\dots ,F^k_i\right)$ is then passed through a multi-layer perceptron^44^ whose output is a latent representation of the earthquake features. This representation is then summed over the past *N* earthquakes, like the sum that defines $\lambda$ in the ETAS model. The encoder is clearly invariant to permutations of catalog rows. Simply put, this encoder essentially mimics the structure of them time-dependent part of an ETAS model, only replacing the function *f* with a neural network, allowing to parameterize a much larger family of functions.
2. *Long range seismicity:* The goal of this encoder is to capture long- and short-term seismicity at the point (*x*, *y*) at time *t*. The features for this model are built as follows. For each such point we calculate *n*(*T*, *d*, *M*), which is the number of earthquakes with magnitude larger than *M*, that occurred at most *T* seconds prior to *t*, at epicentral distance smaller than *d* from (*x*, *y*). For implementation simplicity we use $L_1$ distance, but this choice has negligible effects on the results. The parameters *T*, *d*, *M* are taken from a predefined list. The values of *T* and *d* are logarithmically spaced, allowing to capture very long histories as well as recent activity. This produces a feature vector $(n_1, \dots , n_k)$ per spatial location. Following a weight-sharing strategy similar to that of the recent earthquake encoder, we then use a multi-layer perceptron to parameterize a function *g*(*n*, *T*, *d*, *M*) which is applied to all spatial locations. Implementation details are given in the supplementary material. Our experiments showed that using such weight sharing, i.e. learning a single function *g*, gives significantly better results then learning a more general model that takes the individual $n_i$ as input.
3. *Location:* This encoder is intended to capture local properties for each spatial cell. The model output is a 16-dimensional vector representing the cell’s identity. In Fig. 4 it is seen that the encoding is well correlated with seismicity. The encoder is implemented as a one-hot encoder^44^ (treating every cell as a different class), followed by a single fully connected layer.

### Loss metric

To calculate the loss,

1 $$\begin{aligned} {\mathscr {L}} = \sum _i \log \lambda _i-\iiint \lambda (x,y,t)dx\,dy\,dt \, \end{aligned}$$

we use the method suggested by Omi. et. al^53^. The total train period is divided into intervals that begin an end at the times $\{t_i\}$ where earthquakes occurred. Each training example corresponds to one such interval $[t_i, t_{i+1}]$. For each interval, the catalog of all earthquakes that occurred prior to $t_i$ is passed to the different encoders. The output of the encoders, the latent representation of $H_{t-}$, is then passed to a decoder that outputs $\int _{t_i}^{t_{i+1}}\lambda dt$ for each cell. For this calculation, $\Delta t_i=t_{i+1}-t_{i}$ is supplied as in input to the decoder (see Fig. 1). The second term in Eq. (1) is then evaluated by summing the model output over all examples, and the first term is obtained through automatic differentiation, which is computationally cheap in neural networks.

## Supplementary Information

Supplementary Information.

## Data Availability

The datasets generated and/or analysed during the current study are available in the Japan Meterological Agency (JMA) earthquake catalog, https://www.data.jma.go.jp/svd/eqev/data/bulletin/index_e.html.
