# Supplementary material for: A neural encoder for earthquake rate forecasting
Source: Sci Rep. 2023 Jul 31;13:12350. doi: 10.1038/s41598-023-38033-9 (PMC10390553; doi:10.1038/s41598-023-38033-9)
Supplement: Supplementary file 1 — Supplementary Information. [file 41598_2023_38033_MOESM1_ESM.pdf]

## Supplementary material

### I. BENCMHMARK ETAS MODEL

As a benchmark, we used the FORTRAN implementation of the ETAS model published by Zhuang [1–4]. The model writes the conditional intensity as

$$\lambda(x, y, t) = \mu_0(x, y, t) + \sum_{t_i < t} f(x - x_i, y - y_i, t - t_i, z_i, M_i) \ , \tag{1}$$

where $x, y, t$ are space and time coordinates at which the rate is evaluated. and $x_i, y_i, t_i, z_i, M_i$ are cataloged values of past earthquake's space and time coordinates, depth and magnitude, respectively. The sum is restricted to events that occurred prior to the evaluation time $t$.

| | | **Train** | | **Validation** | | **Test** | | **Total** | |
|---|---|---|---|---|---|---|---|---|---|
| | | 1979-1995 | | 1996-2003 | | 2004-2011 | | 1979-2011 | |
| Region | Magnitude | total | /year | total | /year | total | /year | total | /year |
| A | All | 1238 | 72.8 | 397 | 49.6 | 517 | 64.6 | 2152 | 65.2 |
| | 5 or above | 506 | 29.8 | 135 | 16.9 | 196 | 24.5 | 837 | 25.4 |
| | 6 or above | 49 | 2.9 | 14 | 1.8 | 32 | 4.0 | 95 | 2.9 |
| | 7 or above | 4 | 0.24 | 1 | 0.13 | 7 | 0.88 | 12 | 0.36 |
| B | All | 1686 | 99 | 1159 | 144 | 1027 | 128 | 3872 | 117 |
| | 5 or above | 766 | 45 | 377 | 47 | 336 | 42 | 1479 | 44 |
| | 6 or above | 60 | 3.5 | 39 | 4.9 | 17 | 2.1 | 116 | 3.5 |
| | 7 or above | 2 | 0.12 | 4 | 0.50 | 1 | 0.13 | 7 | 0.21 |
| C | All | 1325 | 78 | 524 | 66 | 592 | 74 | 2441 | 74 |
| | 6 or above | 112 | 6.6 | 54 | 6.8 | 56 | 7.0 | 222 | 6.7 |
| | 7 or above | 7 | 0.41 | 5 | 0.63 | 8 | 1.00 | 20 | 0.61 |

TABLE I. Total number and yearly rates of seismicity in the investigated regions.

### II. DESCRIPTION OF THE ENCODERS

The catalog encoder consists from two separate models, each capturing different properties of earthquake distributions. The first model, described in Section II A, summarizes the contributions of recent earthquakes on the seismicity in the studied domain. It can be thought of as a direct generalization of the $f$ function in Equation (1). The second model, in contrast, describes the seismicity (i.e. the average rate of earthquakes in the past day or month or year) in the studied domain. It is described in Section II B. Additionally, we introduce in Section II C an encoder that does not directly encode earthquakes, but rather, can learn location-dependent weights. It can be thought of as an approximation of the background rate function $\mu$ in Equation (1).

The models below are structured as follows. Define some study region $A$, and a time range $[T_0, T_1]$. Given a location $(x, y) \in A$, a time $t \in [T_0, T_1]$ and a catalog of earthquakes $\Omega$, every model is some function

$E(x, y, t, \Omega) \to \mathbb{R}^n$ (e.g. the long term seismicity model will return a vector of average rates of earthquakes for different regions around $(x, y)$ and time ranges before $t$).

The models have two stages - a **feature calculation stage**, and an **encoding stage**. The feature calculation is a human-engineered function $F(x, y, t, \Omega) \to \mathbb{R}^e$ of real values, named "features". These features represent values that are known to be relevant to the distribution of earthquakes, e.g. one feature we use below is the reciprocal of the time that elapsed since previous earthquakes. The full list of features is given in Section III A.

The encoding stage is some (trainable) machine learned model $M(v) \to \mathbb{R}^n$. So the final encoding function $E(x, y, t, \Omega)$ is $E(x, y, t, \Omega) = M(F(x, y, t, \Omega))$. The architecture of the model can be easily changed in the future, e.g. by using a Recurring Neural Network (RNN) if we wish to capture some temporal connection between the features, or a Convolutional Neural Network (CNN) if we suspect that there is some relationship between spatially adjacent features. Here we use multilayer perceptrons, described in Section III A. The outputs of these models are concatenated, and then used as an input to an inference model. This approach is very versatile – we can add more encoding models, change some of the feature calculation, or replace the architectures – all depending on the task that we want to solve. Simultaneously, we can transfer trained encoders to solve related tasks, as we describe in the main text.

The source code is available at [5].

### A. Recent Earthquakes encoder

The goal of this encoder model is to generalize the $f$ function in Equation (1). In other words, we want to be able to express a sum of some contribution from every earthquake in the (relatively recent) past. We limit ourselves to some $N$ earthquakes in the past ($N$ can be fairly large, covering on average years before $t$).

The feature engineering function $F_{recent}(x, y, t, \Omega)$ in this case outputs a matrix $R_{e\times N}$. Each column in $R_{e\times N}$ corresponds to a single earthquake (out of the past $N$ earthquakes). Denote the location, depth, time and magnitude of a past earthquake by $x_j, y_j, z_j, t_j$ and $M_j$, respectively, $1 \le j \le N$. We choose some $e$ functions $f_i(x, y, t, x_j, y_j, z_j, t_j, M_j) \to \mathbb{R}$ for $1 \le i \le e$, that calculate values that are known to be useful for estimating the target output. Every row in $R_{e\times N}$ corresponds to a function, meaning that $R_{i,j} = f_i(x, y, t, x_j, y_j, z_j, t_j, M_j)$. Unlike the ETAS $f$, these are non-parametric functions – the parameterization is replaced with the encoding stage. Specifically, for the application in Section III, the feature functions are the building blocks of the ETAS $f$ function. They include the exponent of the magnitude, $f_1 = e^{M_j}$, the reciprocal of elapsed times $f_2 = (t - t_j)^{-1}$, etc. The full list appears in Section III A. In such applications there is usually little harm in adding more feature functions, and testing whether they improve the performance of the model.

The encoder we choose is invariant to the order of the input earthquakes. Firstly, this closely mimics the behavior of the sum in ETAS. Additionally, this has the advantage that a small change in the input earthquakes won't change the encoding significantly. We achieve this order invariance by using a series of convolutional layers with shapes $(1, e_j)$ and $e_{j+1}$ filters per layer (here $e_0 = e$), and then summing along the earthquake axis.

### B. Seismicity Rate encoder

Here the goal is to capture long- and short-term seismicity in the study region - the average rate of earthquakes per day or per month, before $t$ and around $(x, y)$. Intuitively, if the region around $(x, y)$ had recently seen a lot of seismicity, it is natural to predict that there will be a lot of seismic activity there in the near future as well.

The straightforward way to build features for this model is as follows. Define a set of time look-back thresholds $\{b_i\}_{i=1}^{B}$, a set of distance thresholds $\{d_j\}_{j=1}^{D}$, and a set of magnitude thresholds $\{m_k\}_{k=1}^{M}$. Then, given an input space-time $(x, y, t)$, for every $1 \le i \le B$, $1 \le j \le D$ and $1 \le k \le M$, calculate $n_{i,j,k}(x, y, t)$

[$h$]

FIG. 1. Schematic of the Recent earthquakes encoder. We encode each earthquake separately, using some feature engineering functions. We then apply a trainable function on every encoded earthquake, and sum the results.

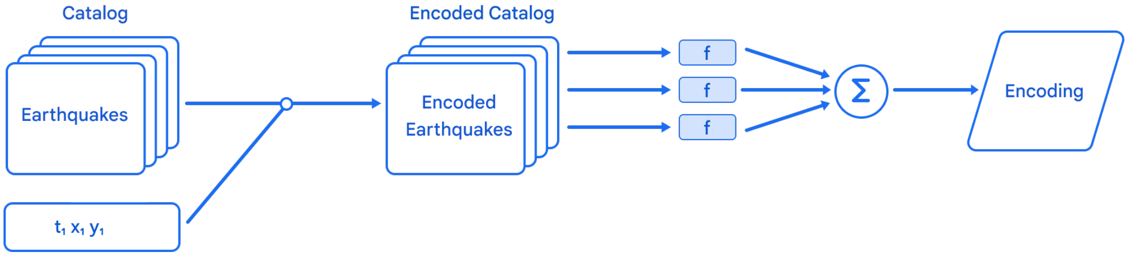


[$h$]

FIG. 2. Schematic of the Seismicity encoder. We calculate average rates of earthquakes for different time ranges, magnitude thresholds, and regions around the input point. We then apply a trainable function, that uses weight sharing in order to infer the impact of rare (large) earthquakes from frequent (smaller) earthquakes.

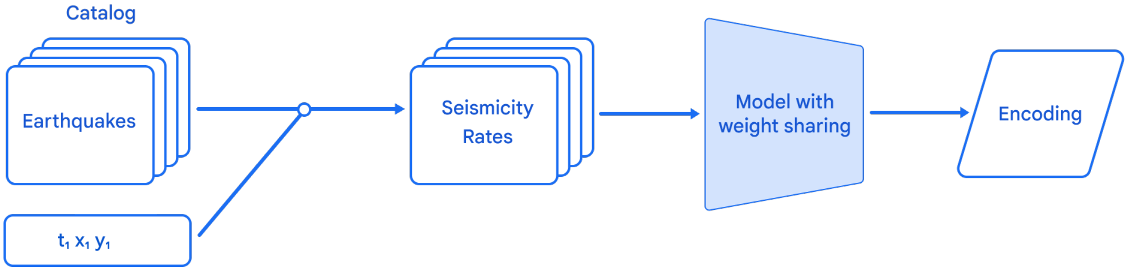


– the number of earthquakes in the $b_i$ seconds prior to $t$, that are of magnitude at least $m_k$ and are in the (axis-aligned) square with side $d_i$ meters centered in $(x, y)$.

We could then take a vector of all $n_{i,j,k}(x, y, t)$ and use some model to encode them. However, note what happens for small values of $b_i$, $d_j$, and large values of $m_k$. For such values, it is possible that we do not see many examples of $(x, y, t)$ in the training set for which $n_{i,j,k}(x, y, t) > 0$. However, we know that if $n_{i,j,k}(x, y, t) > 0$ for some small $b_i$, $d_j$, and a large $m_k$, then it greatly increases the future rate of earthquakes near $(x, y)$. In order to help the model infer from values of $b_i$, $d_j$, $m_k$ that often have $n_{i,j,k}(x, y, t) > 0$ to ones where $n_{i,j,k}$ tends to be low (or 0), we add more features and a specific encoder.

The feature engineering function $F_{seismicity}(x, y, t, \Omega)$ outputs a matrix $S_{u\times B\cdot D\cdot M}$. Each column corresponds to a single choice of $b_i$, $d_j$ and $m_k$. The first row holds the values $n_{i,j,k}(x, y, t)$. From the second row onward the values are functions $g_s(b_i, d_j, m_k)$ for $1 \leq s \leq u - 1$. For instance, the second row can hold the exponent of $m_k$ (that correspond to the column), the third row can hold the reciprocal of $b_i$, and so on. Again, the functions are building blocks inspired by ETAS, and there is little harm in adding more feature functions. The full list of feature functions appears in Section III A.

In order to infer from some values of $b_i$, $d_j$, $m_k$ to others, we use a similar architecture to the one used in Section II A. We start by applying - some $r$ 1-column convolutional layers with shapes $(1, u_j)$ and $u_{j+1}$ filters per layer (here $u_0 = u$ and $0 \leq j \leq r - 1$). Thus we learn a set of $u_r$ functions $h_t(x, y, t, n_{i,j,k}(x, y, t), b_i, d_j, m_k)$, $1 \leq t \leq u_r$. Note that the functions are shared for different values of $b_i, d_j, m_k$, even those where $n_{i,j,k}(x, y, t)$ is often equal to 0. We then flatten the resulting $u_r \times B \cdot D \cdot M$ matrix into a single vector, and apply some fully connected layers to combine these functions.

### C. Location encoder

The final encoders attempts to capture the general spatial correlation between the location $(x, y)$ of the example and the target output. We divide the study region $A$ into a regularly spaced grid and represent the location $(x, y)$ with a 1-hot encoding of the grid cell that contains $(x, y)$. The encoding stage is simply a single fully connected layer applied on the 1-hot encoding.

## III. RATE PREDICTION

Our first goal is to build a model that would output the conditional intensity function $\lambda(x, y, t|H_t)$ of earthquakes in $A$ above some threshold magnitude $M_0$, similarly to the output of ETAS. We call this model FERN (Forecasting Earthquake Rates with Neural nets). FERN is a parametric model, and we sometimes write $\text{FERN}_\theta$ to emphasize that $\theta$ is some assignment to the various weights in the model (both in the encoders that were described above, and in the head model described below).

The output of ETAS is notoriously difficult to integrate numerically. Assuming that our model will produce a similar output, we wish to avoid numerical integration when calculating the log-likelihood loss. We therefore follow the architecture in [6], by introducing a 'lead time' $\Delta t$, and outputting:

$$\text{FERN}(x, y, t, \Delta t) = \int_t^{t+\Delta t} \lambda(x, y, \tau|H_{t-})d\tau.$$

Precisely as described in [6], we can calculate $\lambda(x, y, t|H_{t-})$ by using back-propagation to the input $\Delta t$. Also following [6], the head model is a series of fully connected layers with monotonically increasing activation functions ($tanh$) and non-negative weights – thus we achieve that the back-propagation of the output to the input $\Delta t$ is non-negative.

The loss function $\mathcal{L}$ that we optimize for is (an approximation of) the negative of the log-likelihood of the observed catalog under the conditional intensity function the is produced by the model $\text{FERN}_\theta(x, y, t, \Delta t)$. Formally, denote by $\{(x_i, y_i)\}_{i=1}^n$, $\{t_i\}_{i=1}^n$, $\{m_i\}_{i=1}^n$ the locations, times and magnitudes (respectively), of all $n$ earthquakes in the target region (i.e. $(x_i, y_i) \in A$, $t_i \in [T_0, T_1]$ and $m_i \geq M_0$. Then:

$$\mathcal{L}(\theta) = -\log L(\{(x_i, y_i, t_i)\}_{i=1}^n) = \sum_{i=1}^n \log \lambda(x_i, y_i, t_i|H_{t_i-}) - \int_{T_0}^{T_1} \iint_A \lambda(x, y, t|H_{t-})dxdydt.$$

Define $(n + 1) \cdot q$ training examples, where $q$ is some constant (and $n$ is the number of target earthquakes in the training set, as before). The examples are denoted by $e_{i,j}$, for $1 \leq i \leq n + 1$ and $1 \leq j \leq q$. In order to calculate this loss during training each example $e_{i,j}$ is responsible for calculating a part of this formula - the loss of the example $\mathcal{L}(e_{i,j}|\theta)$. As shown below, the sum of the contributions of all examples $\mathcal{L}(e_{i,j}|\theta)$ approximates the log likelihood $\mathcal{L}(\theta)$.

We construct the examples as follows. Extend the series $\{t_i\}_{i=1}^n$ by adding $t_0 = T_0$ and $t_{n+1} = T_1$. For every example $e_{i,j}$, define the 'history time' $h_i = t_{i-1}$, 'lead time' $\ell_i = (t_i - t_{i-1})$, and 'location' $(a_{i,j}, b_{i,j}) \in A$. Set $(a_{i,1}, b_{i,1}) = (x_i, y_i)$. For $2 \leq j \leq q$, the locations $(a_{i,j}, b_{i,j})$ can be arbitrary, but their choice can impact the accuracy of the approximation of $\mathcal{L}(\theta)$. In our implementation we choose $(a_{i,j}, b_{i,j})$ to be evenly spaced from $(x_i, y_i)$ on a regular grid in $A$.

We can define:

$$\mathcal{L}(e_{i,j}|\theta) = \begin{cases} \log \lambda(a_j, b_j, h_i + \ell_i|H_{h_i}) - \int_{h_i}^{h_{i+1}} \lambda(a_j, y_j, \tau|H_{h_i})d\tau & \text{, if } j = 1 \\ -\int_{h_i}^{h_{i+1}} \lambda(a_j, y_j, \tau|H_{h_i})d\tau & \text{, otherwise.} \end{cases}$$

Now, we assume that, as in ETAS, $\lambda(x_i, y_i, t_i|H_{t_i-}) = \lambda(x_i, y_i, t_i|H_{t_{i-1}})$ (meaning that the events that happen since the last target earthquake do not affect the output). Note therefore that if we sum $\mathcal{L}(e_{i,j}|\theta)$ over all examples $e_{i,j}$, we get exactly $\sum_{i=1}^n \log \lambda(x_i, y_i, t_i|H_{t_i-})$ from the first case. As for the integral part, when summing $\mathcal{L}(e_{i,j}|\theta)$ over all $1 \leq j \leq q$, we get an approximation of $-\int_{t_{i-1}}^{t_i} \iint_A \lambda(x, y, t|H_{t-})dxdydt$. Summing these integrals for $1 \leq i \leq n + 1$ gives us the desired approximation of $\mathcal{L}(\theta)$.

[$h$]

FIG. 3. Schematic of FERN.

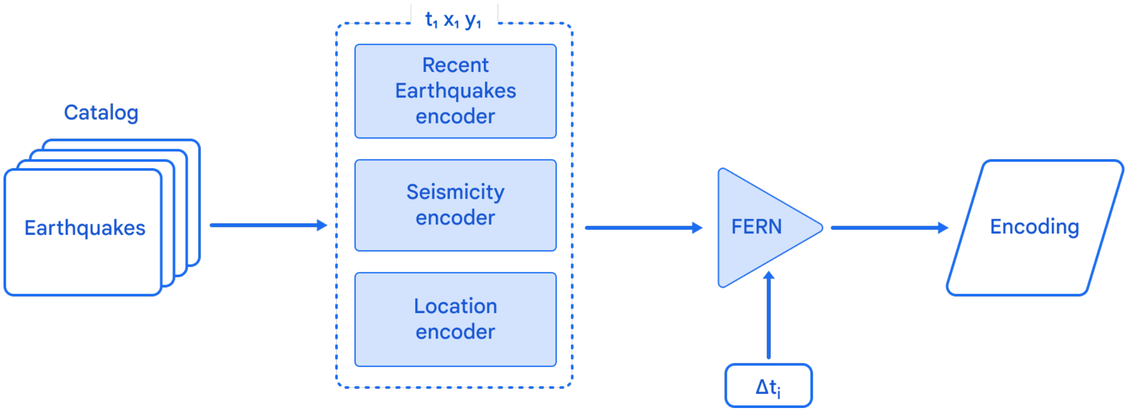


### A. Method and architecture

In order to produce the results that we report in the main text, we performed the following procedure for all three study regions:

1. Select non-overlapping training period, validation period and test period.
2. Train numerous models using the training period, and evaluate them on the validation period. The runs differ by the sizes of the layers in every encoder and in the FERN head model, by the regularization we apply for every layer, by the learning rate, and other hyperparameters.
3. Select good hyperparameters using the validation set. Good hyperparameters give the best validation loss, while the training and validation curves being relatively smooth.
4. Retrain the model using the same hyperparameters on both the training and validation periods. Since there is randomness in the training, train 20 models using these hyperparameters, and select one with the median train loss. Report the log-likelihood on the test period.

In practice, we can evaluate the log-likelihood of the test set with a finer spatial grid (taking a larger value for $q$) that what was used for training. Thus we get a more accurate log-likelihood score, while keeping the training relatively faster. The values for the log-likelihood that we report below are achieved with such an evaluation.

In the table below we specify all of the values that were chosen for our final reported results. We have 3 regions A, B, C (described in the main text), and 2 models per region, which we call FERN and FERN$^+$. In FERN the input catalog only contains the target earthquakes. In FERN$^+$ the input catalog also contains smaller earthquakes, with a magnitude threshold that is termed 'feature magnitude'. Note that the set of examples $e_{i,j}$ is the same for FERN and FERN$^+$, the difference between them is in the richness of the encoding (i.e. they have different history objects).

All of the regularization that are specified above are L2 regularization, per encoder model or head model. The optimizer we use is Adam with the learning rate that is specified in the table. Additionally, we decrease the learning rate by a multiplicative constant that is specified in the 'learning rate decay' row.

The following feature engineering functions were used:

## IV. ONE-DAY FORECASTING

In order to demonstrate the the encoders learned some latent representation of the state of seismicity in the trained region, we want to reuse them for a different (related) task. The task we chose is 1-day forecasting.

| | Region A | | Region B | | Region C | |
|---|---|---|---|---|---|---|
| | FERN | FERN$^+$ | FERN | FERN$^+$ | FERN | FERN$^+$ |
| Target magnitude | 4.5 | | | | 5 | |
| Feature magnitude | 4.5 | 3 | 4.5 | 3 | 5 | 3 |
| Longitude range | (141, 145) | | (145, 154) | | (141, 154) | |
| Latitude range | (36, 42) | | (42, 47) | | (36, 47) | |
| Maximum depth | 100 | | | | | |
| Batch size | 64 | | | | | |
| Epochs | 100 | | | | | |
| Learning rate | 3e-5 | 4e-5 | 2e-5 | 4e-5 | 1e-5 | 4e-5 |
| Learning rate decay | 0.98 | 0.98 | 0.99 | 0.99 | 1 | 1 |
| Recent Earthquake regularization | 1e-4 | 1e-3 | 1e-3 | 1e-3 | 1e-5 | 1e-5 |
| Seismicity Rate regularization | 1e-4 | 1e-4 | 1e-4 | 1e-4 | 1e-3 | 1e-2 |
| Location regularization | 1e-4 | 1e-4 | 1e-4 | 1e-4 | 1e-5 | 1e-4 |
| FERN regularization | 1e-3 | 1e-3 | 1e-5 | 1e-5 | 1e-4 | 1e-4 |
| $q$ for the example grid | 600 | | 1125 | | 572 | |
| $N$ for Recent Earthquakes | 40 | | | | | |
| $\{b_i\}_{i=1}^{B}$ for Seismicity Rate | 5 $[m]$, 1 $[h]$, 1 $[d]$, 7 $[d]$, 30 $[d]$, 90 $[d]$, 1 $[y]$ | | | | | |
| $\{d_j\}_{j=1}^{D}$ for Seismicity Rate | 1, 2, 4, ..., 256 $[km]$ | | | | 1, 2, 4, ..., 512 $[km]$ | |
| $\{m_k\}_{k=1}^{M}$ for Seismicity Rate | 4.5, 5, 5.5, 6 | 3, 4, 5, 6 | 4.5, 5, 5.5, 6 | 3, 3.5, 4, ..., 6 | 5, 5.5, 6, 6.5, 7 | 3, 3.5, 4, ..., 7 |
| Grid size for the Locations encoder | $0.2 \times 0.2$ $[deg^2]$ | | | | $0.5 \times 0.5$ $[deg^2]$ | |
| CNN filter sizes for Recent Earthquakes | 16, 8, 4 | | | | | |
| CNN filter sizes for Seismicity Rate | 16, 8 | | | | | |
| Layer sizes for Seismicity Rate | 16 | | | | | |
| Layer sizes for the Locations encoder | 16 | | | | | |
| Layer sizes for FERN | 64, 1 | | | | | |

TABLE II. Hyper parameters for all neural networks used in the manuscript.

Recent earthquakes features

| | $f(x, y, t, x_j, y_j, z_j, t_j, M_j)$ |
|---|---|
| $f_1$ | $\left\|\left\|(x - x_j, y - y_j)\right\|\right\|^{-1}$ |
| $f_2$ | $\log\left(\left\|\left\|(x - x_j, y - y_j)\right\|\right\|\right)$ |
| $f_3$ | $x - x_j$ |
| $f_4$ | $y - y_j$ |
| $f_5$ | $e^{M_j}$ |
| $f_6$ | $e^{-M_j}$ |
| $f_7$ | $\log(z_j)$ |
| $f_8$ | $z_j$ |
| $f_9$ | $\log(t - t_j)$ |
| $f_{10}$ | $(t - t_j)^{-1}$ |

Seismicity rate features

| | $g(b_i, d_j, m_j)$ |
|---|---|
| $g_1$ | $e^{m_k}$ |
| $g_2$ | $e^{-m_k}$ |
| $g_3$ | $b_i^{-1}$ |
| $g_4$ | $\log(b_i)$ |
| $g_5$ | $d_j^{-1}$ |
| $g_6$ | $\log(d_j)$ |

TABLE III. Feature functions for the recent earthquakes and seismicity rate encoders.

Specifically, given the history up to the end of one day, the model should output the expected number of earthquakes in spatial bins for the following day. The standard way to get such forecasts using ETAS is by sampling 100,000 simulated catalogs (per day) from the ETAS conditional intensity function, and then averaging the number of predicted earthquakes per cell. This task has a classical form of a machine learning regression problem, since we have labels – the actual number of earthquakes that occurred in every bin.

Here we choose to forecast in $0.5 \times 0.5$ $[deg^2]$ cells. Additionally, we do not have magnitude bins. So, our labels and forecasts can be denoted as $\Omega = \{\omega_{i,j} | i \in T, j \in S\}$ and $\Lambda = \{\lambda_{i,j} | i \in T, j \in S\}$ (respectively), where $T$ is the set of indices of days, and $S$ is the set of indices of grid cells.

We compare between the performance of ETAS and FERN (and FERN$^+$) by calculating common metrics - L-test, S-test and N-test ([7]). Note that often these metrics are evaluated by comparing the score that a forecast is getting for the actual observed catalog, against catalogs that were sampled from the forecast (assuming that the forecast is actually reporting the Poisson rate of earthquakes in every bin). This is a way of evaluating how good a forecast is without competing models. We prefer to compare between models, and therefore we do not do this simulation step. Instead, we calculate the different metrics for the three competing models, and compare them against each other. Additionally, we calculate the scores for two simple models – a 'Poisson' model, which learns a constant rate of earthquakes per spatial bin, and a 'Perfect' model, which always outputs the correct output (that is, $\lambda_{i,j}^{Perfect} = \omega_{i,j}$). These models give us a scale for the difference between ETAS, FERN and FERN$^+$.

- **L-test**: the log-likelihood of the observed catalog, under the assumption that the model is outputting Poisson rates per bin:

$$\text{L-test}(\Omega|\Lambda) = \sum_{i \in T, j \in S} (\omega_{i,j} \cdot \log(\lambda_{i,j}) - \lambda_{i,j}).$$

- **S-test**: the log-likelihood of the spatial distribution of the observed catalog, under the assumption that the average output of the model per spatial bin is a Poisson rate:
Define $N_{obs} = \sum_{i \in T, j \in S} \omega(i,j)$ and $N_{fore} = \sum_{i \in T, j \in S} \lambda(i,j)$, which are the total numbers of observed and forecasted earthquakes (respectively). Denote the spatial distributions $\Omega^S = \{\omega_j^S | j \in S\}$ and $\Lambda^S = \{\lambda_j^S | j \in S\}$, where:

$$\omega_j^S = \sum_{i \in T} \omega_{i,j}, \text{ and } \lambda_j^S = \frac{N_{obs}}{N_{fore}} \sum_{i \in T} \lambda_{i,j}$$

Then the score is:

$$\text{S-test}(\Omega^S|\Lambda^S) = \sum_{j \in S} (\omega_j^S \cdot \log(\lambda_j^S) - \lambda_j^S).$$

- **N-test**: the probability of observing the actual number of earthquakes, under the assumption that the model is outputting Poisson rates per bin. We can calculate the cumulative distribution of the number of earthquakes:

$$F(x|N_{fore}) = \exp(-N_{fore}) \cdot \sum_{i=0}^{x} \frac{(N_{fore})^i}{i!}.$$

The N-test metrics are the probabilities of observing at least $N_{obs} - 1$ earthquakes and at most $N_{obs}$ earthquakes:

$$\delta_1 = 1 - F(N_{obs} - 1|N_{fore}) \text{ and } \delta_2 = F(N_{obs}|N_{fore}).$$

In all of our models (FERN and FERN$^+$, for three regions), we take the trained encoders, and use them to calculate latent representations for the state of seismicity (by inputting the time of the end of a day, and

the location of the center of a spatial bin). Then we train a simple fully connected model to map this latent representation to the $\Omega$ labels, optimizing for the standard Poisson loss (which is equivalent to optimizing for the L-test). We use the same four step procedure that is described in Section III A in order to select the best model. The chosen hyperparameters appear in the table below:

| | Region A | | Region B | | Region C | |
|---|---|---|---|---|---|---|
| | FERN | FERN$^+$ | FERN | FERN$^+$ | FERN | FERN$^+$ |
| Batch size | 256 | | | | | |
| Epochs | 200 | | | | | |
| Learning rate | 7e-4 | 6e-4 | 8e-4 | 5e-4 | 9e-4 | 9e-4 |
| Head regularization | 5e-7 | 4e-7 | 3e-8 | 3e-7 | 4e-8 | 3e-8 |


[1] J. Zhuang, Y. Ogata, and D. Vere-Jones, Stochastic declustering of space-time earthquake occurrences, Journal of the American Statistical Association **97**, 369 (2002).

[2] J. Zhuang, Y. Ogata, and D. Vere-Jones, Analyzing earthquake clustering features by using stochastic reconstruction, Journal of Geophysical Research: Solid Earth **109** (2004).

[3] J. Zhuang, Second-order residual analysis of spatiotemporal point processes and applications in model evaluation, Journal of the Royal Statistical Society: Series B (Statistical Methodology) **68**, 635 (2006).

[4] J. Zhuang, Next-day earthquake forecasts for the japan region generated by the etas model, Earth, planets and space **63**, 207 (2011).

[5] O. Zlydenko and et. al., Source code will be available upon acceptance, github (2022).

[6] T. Omi, N. Ueda, and K. Aihara, Fully neural network based model for general temporal point processes, in *Advances in Neural Information Processing Systems 32: Annual Conference on Neural Information Processing Systems 2019, NeurIPS 2019, December 8-14, 2019, Vancouver, BC, Canada*, edited by H. M. Wallach, H. Larochelle, A. Beygelzimer, F. d'Alché-Buc, E. B. Fox, and R. Garnett (2019) pp. 2120–2129.

[7] D. Schorlemmer, M. C. Gerstenberger, S. Wiemer, D. D. Jackson, and D. A. Rhoades, Earthquake likelihood model testing, Seismological Research Letters **78**, 17 (2007).
